# Post-treatment de-phosphorylation of p53 correlates with dasatinib responsiveness in malignant melanoma

**DOI:** 10.1186/s12860-018-0180-1

**Published:** 2018-12-27

**Authors:** Josip Skoko, Jan Rožanc, Emilie M. Charles, Leonidas G. Alexopoulos, Markus Rehm

**Affiliations:** 1 Department of Physiology & Medical Physics, Royal College of Surgeons in Ireland, Dublin 2, Ireland; 2 Centre for Systems Medicine, Royal College of Surgeons in Ireland, Dublin 2, Ireland; 3 Institute of Cell Biology and Immunology, University of Stuttgart, Allmandring 31, 70569 Stuttgart, Germany; 4 ProtATonce Ltd, Science Park Demokritos, Athens, Greece; 5 Department of Mechanical Engineering, National Technical University of Athens, Athens, Greece; 6 Stuttgart Research Center Systems Biology, University of Stuttgart, Stuttgart, Germany

**Keywords:** Melanoma, Dasatinib, Dacarbazine, Proliferation, Cell death

## Abstract

**Background:**

Dasatinib (Sprycel) was developed as a tyrosine kinase inhibitor targeting Bcr-Abl and the family of Src kinases. Dasatinib is commonly used for the treatment of acute lymphoblastic and chronic myelogenous leukemia. Previous clinical studies in melanoma returned inconclusive results and suggested that patients respond highly heterogeneously to dasatinib as single agent or in combination with standard-of-care chemotherapeutic dacarbazine. Reliable biomarkers to predict dasatinib responsiveness in melanoma have not yet been developed.

**Results:**

Here, we collected comprehensive in vitro data from experimentally well-controlled conditions to study the effect of dasatinib, alone and in combination with dacarbazine, on cell proliferation and cell survival. Sixteen treatment conditions, covering therapeutically relevant concentrations ranges of both drugs, were tested in 12 melanoma cell lines with diverse mutational backgrounds. Melanoma cell lines responded heterogeneously and, importantly, dasatinib and dacarbazine did not synergize in suppressing proliferation or inducing cell death. Since dasatinib is a promiscuous kinase inhibitor, possibly affecting multiple disease-relevant pathways, we also determined if basal phospho-protein amounts and treatment-induced changes in phospho-protein levels are indicative of dasatinib responsiveness. We found that treatment-induced de-phosphorylation of p53 correlates with dasatinib responsiveness in malignant melanoma.

**Conclusions:**

Loss of p53 phosphorylation might be an interesting candidate for a kinetic marker of dasatinib responsiveness in melanoma, pending more comprehensive validation in future studies.

## Background

Dasatinib (N-(2-chloro-6-methyl-phenyl)-2-((6-(4-(2-hydroxyethyl)-piperazin-1-yl)-2-methylpyrimidin-4-yl)amino)-1,3-thiazole-5-carboxamide) is an orally active tyrosine kinase inhibitor developed to target Bcr-Abl and the family of Src kinases [1]. Dasatinib is used as a first or second line treatment in patients with chronic myeloid leukemia as well as a second line treatment in patients suffering from Philadelphia-positive acute lymphoblastic leukemia [2]. While initially thought to be rather specific in its target selectivity, subsequent comparative kinase inhibitor and kinome targeting screens highlighted that dasatinib also blocks various other kinases at therapeutically relevant concentrations [3–6]. Among these targets are, for example, focal adhesion kinase (FAK), Bruton’s tyrosine kinase (BTK), various ephrin receptors (EPHs), as well as epidermal growth factor receptor (EGFR) and p38 mitogen-activated protein kinase (p38 MAPK) [3–5, 7]. Largely in line with its intended target specificity, dasatinib treatment primarily suppresses cell growth and proliferation [1]. Secondary to growth arrest, dasatinib treatment can induce programmed cell death, with apoptosis being the primary death modality [8]. The molecular mechanisms by which dasatinib treatment results in apoptosis induction, however, remain incompletely understood.

Dasatinib has been and still is being tested as a single agent and in combination therapies, for example together with other kinase inhibitors or genotoxic chemotherapeutics, in haematological cancers and solid tumours. At the time of writing, clinicaltrials.gov lists 72 active and planned studies. In melanoma, the therapeutic efficacy of dasatinib was tested as a single treatment as well as in combination with the standard-of-care chemotherapeutic dacarbazine (DTIC) in phase I/II trials. In a phase I trial with 29 patients, the reported objective response rate (ORR) was 13.8%, and the 6-month progression-free survival (PFS) was 20.7%. In a phase II trial with 36 patients, the ORR was 5%, and the 6-month PFS was 13%. [9, 10]. With the arrival of novel targeted therapeutics, such as MEK or BRAFV600 inhibitors as well as immune checkpoint inhibitors [11], studies on dasatinib in melanoma have ceased. While a number of melanoma patients benefited from dasatinib treatment, it still remains largely unknown what determines or is indicative of responsiveness, and if the combination of dasatinib with dacarbazine could possibly improve overall treatment responses. Since the majority of melanoma patients in poorly funded healthcare environments to this day still do not have routine access to costly BRAFV600/MEK inhibitor or immunotherapeutics based treatments, these patients still rely on chemotherapy. Chemotherapy also remains the standard-of-care last line treatment. It is therefore warranted not to lose sight of strategies to enhance chemotherapy responsiveness in melanoma. Here, we therefore systematically explored the efficacy of dasatinib and the DNA-alkylating agent dacarbazine, alone and in combination, in impairing melanoma cell proliferation and in inducing cell death in a diverse panel of melanoma cell lines. In addition, we studied if pre-treatment phospho-protein amounts as well as their treatment-induced changes could serve as indicators for treatment responsiveness.

## Results

### Dasatinib impairs proliferation of melanoma cell lines but does not synergise with standard-of-care chemotherapeutic dacarbazine

Dasatinib and dacarbazine both were reported to impair cell proliferation, either by kinase inhibition or by DNA damage-induced cell cycle arrest [8, 12]. To obtain a detailed overview of the potency of both drugs in affecting cell proliferation, when administered alone or in combination, we performed comprehensive measurements of cell counts, using flow cytometry. Twelve melanoma cell lines with varying mutation backgrounds (see Materials and methods) were treated with dasatinib and dacarbazine at 16 different conditions each. The concentration range for dasatinib was chosen along concentrations achieved in patient serum (up to > 100 nM) [3, 4, 13]. The maximum concentration of dacarbazine (1 mg/ml) was chosen based on amounts achievable in patient serum during systemic therapy (29 μg/ml) and amounts achieved for DNA-alkylating agents during isolated limber perfusion/infusion [14, 15].

**Fig. 1:**
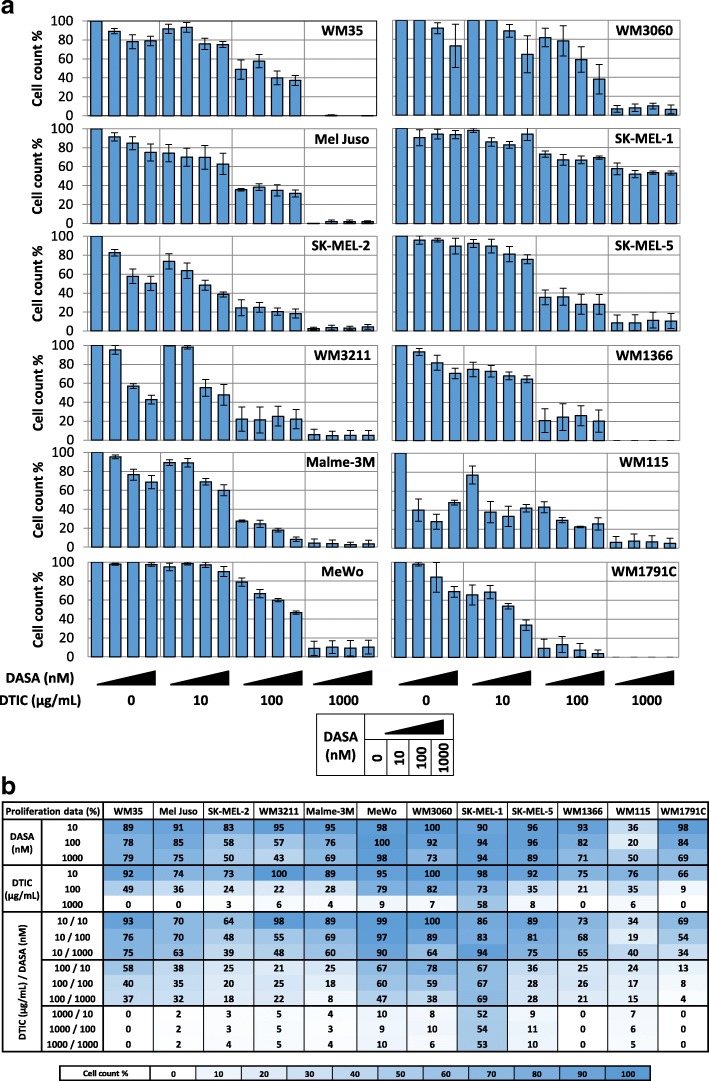
Heterogeneous growth arrest in melanoma cells treated with dasatinib, alone or in combination with dacarbazine. **a** Cell proliferation (cell count) of melanoma cell lines after single and combination treatment with dasatinib and dacarbazine (72 h). Bars represent mean values of percentage change normalized to the control. Data are from three independent experiments, each run with triplicate samples. Error bars show SEM. **b** Summary table of cell proliferation data from (**a**)

72 h after treatment commenced, heterogeneous responses to dasatinib or dacarbazine on cell proliferation were observed. For dasatinib single agent treatment, WM115 was the most sensitive cell line, with 10–100 nM sufficient to reduce proliferation to approximately 25% (Fig. 1a and b). In SK-MEL-2 and WM3211 cells, similar concentrations reduced proliferation to only 50%. In all other cell lines clinically irrelevant concentration up to 10 μM were required to achieve comparable effects on growth arrest (not shown), with the exception of SK-MEL-1 cells, which remained resistant even at this concentration. For dacarbazine responsiveness, we observed that a concentration of 10–100 μg/ml reduces proliferation in various cell lines to approximately 25%, including SK-MEL-2, WM3211, WM1366, Malme-3 M cells (Fig. 1a and b). WM1791C were particularly responsive to dacarbazine, with only 10% proliferation observed at a concentration of 100 μg/ml. As observed for dasatinib treatment, SK-MEL-1 cells were the most resistant cells also upon dacarbazine treatment (Fig. 1a and b). We likewise tested the efficacy of combination treatments of dasatinib and dacarbazine. However, we failed to detect any notable response synergies or drug effect potentiation that manifested in impaired cell proliferation (Fig. 1a and b).

Taken together, we therefore conclude that the responsiveness to dasatinib is heterogeneous across melanoma cell lines. Furthermore, and in contrast to a previous study that suggested dasatinib could synergize with DNA-alkylating agents in melanoma cells [16], we conclude that the combination of dasatinib and dacarbazine does not provide any benefit in inhibiting cell proliferation.

### Dasatinib is ineffective in inducing melanoma cell death, alone or in combination with dacarbazine

**Fig. 2:**
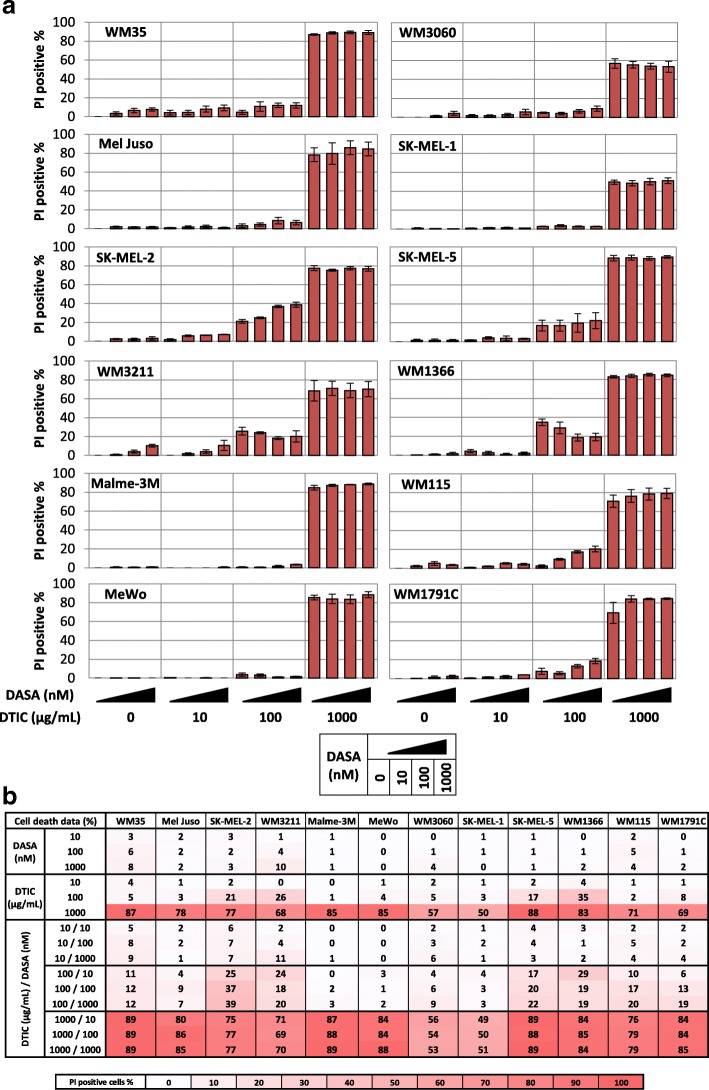
Heterogeneous cell death induction in melanoma cells treated with dasatinib, alone or in combination with dacarbazine. **a** Cell death responsiveness (propidium iodide positivity) of melanoma cell lines after single and combination treatment with dasatinib and dacarbazine (72 h). Bars represent mean values from three independent experiments, each run with triplicate samples. Error bars show SEM. **b** Summary table of cell death data from (**a**)

Clinical studies in which metastatic melanoma patients received dasatinib, as single agent or in combination with dacarbazine, indicated that subgroups of patients responded to these treatments with tumour regression or prolonged survival [9, 10]. It is therefore conceivable that beyond the cytostatic effects of these drugs, prolonged cell cycle arrest might translate into the induction of cell death. Complementary to the readouts in Fig. 1, we therefore determined cell death by uptake of propidium iodide, after single or combination treatment with dasatinib and dacarbazine. Dasatinib as a single agent largely failed to induce cell death at clinically relevant concentrations (Fig. 2a and b). At higher concentrations (10 μM), modest responsiveness of no more than 22–26% cell death could be observed in Mel-Juso and SK-MEL-2 cells (not shown). In contrast, dacarbazine efficiently induced cell death at the highest concentration tested (1 mg/ml), with only WM3060 and SK-MEL-1 cells remaining modestly resistant (approximately 50% cell death) (Fig. 2a and b). We did not identify any synergies in cell death induction for the combination of dasatinib and dacarbazine.

Taken together, we therefore conclude that the cytostatic effects of dasatinib do not necessarily translate into cell death in vitro. Where cell death was observed in response to dasatinib, cell killing was moderate at best. Importantly, the combination with standard of care chemotherapeutic dacarbazine did not induce response synergies or potentiations.

### Post-treatment de-phosphorylation of p53, but not pre-treatment amounts P-p53 or other key phosphoproteins, distinguish dasatinib-responsive from dasatinib-resistant cell lines

Even though dasatinib was developed as a targeted Src/Abl kinase inhibitor, neither Src nor phopho-Src amounts correlate with dasatinib responsiveness in melanoma [17]. While having high affinity for Src, dasatinib also inhibits a number of additional kinases at therapeutically relevant concentrations [3–6]. The efficacy of dasatinib in inducing growth arrest might therefore depend on the basal activities of a multitude of kinase pathways and signalling cascades involved in the control of cell growth and proliferation. Further complications arise from the intricate interplay of multiple kinase signalling networks, so that identifying patterns predictive for dasatinib treatment might require the analysis of multiple phosphorylated proteins. We therefore tested if basal expression amounts of various phospho-proteins or their treatment-induced changes would be suitable as marker candidates to predict the likelihood of dasatinib responsiveness.

To investigate if basal activities of kinase-dependent signal transduction pathways could indicate dasatinib responsiveness in melanoma cells, we investigated phosphoprotein amounts in our panel of cell lines. The amounts of phosphoproteins were then used to test if these allowed separating responder and non-responder cell lines. Targets selected for these studies included upstream kinases, kinases situated further downstream in signal transduction cascades, as well as terminal effectors in the form of selected phospho-regulated transcription factors. All phospho-proteins studied are directly or indirectly involved in the regulation of cellular growth, cell cycle progression and proliferation. In particular, we studied phosphorylated focal adhesion kinase 1 (FAK1 (Y397)), a target associated with c-Src signaling, directly affected by dasatinib and also known for being implicated in downstream RAS/RAF/MEK/ERK signaling, thereby affecting cell growth and proliferation [3, 18]; phosphorylated tyrosine-protein phosphatase non-receptor type 11 (PTN11 (Y542)), belonging to the protein tyrosine phosphatase family and playing roles in cell growth and mitosis control, reported to stimulate the MAPK signaling cascade and acting downstream of various receptor tyrosine kinases [19]; phosphorylated dual specificity mitogen-activated protein kinase kinase-1 (MEK1 (S217/S221)), an kinase centrally involved in the RAS/RAF/MEK/ERK cascade, driving cell proliferation and a major target for novel precision therapeutics for the clinical management of metastatic melanoma [20]; phosphorylated mitogen-activated protein kinase-3 (ERK1 (T202/Y204)), an essential downstream effector of the MAP kinase signal transduction pathway, regulating cell proliferation, differentiation and cell cycle progression and found dysregulated in majority of melanomas [21]; phosphorylated RAC-alpha serine/threonine-protein kinase (AKT1 (S473)), which promotes growth and proliferation downstream of receptor tyrosine kinases and phosphoinositide 3-kinase signalling, but acting upstream of IκB proteins [22]; phosphorylated serine/threonine-protein kinase WNK1 (WNK (T60)), which previously has been studied mostly in the context of kidney function but also appears to have roles in tumorigenesis and cellular proliferation [23]; phosphorylated mitogen-activated protein kinase 9 (JNK2 (T202/Y204)), a kinase embedded into the MAPK cascade, reported to interact with p53 and involved in the regulation of cell proliferation [24]; phosphorylated transcription factor AP-1 (c-JUN (S63)), which is a target of JUN-kinase, involved in stress response signaling and implicated in the regulation of cell cycle progression [25]; phosphorylated NF-κB inhibitor alpha (IκBα (S32/S36)), where phosphorylation induces the proteasomal degradation of IκBα and thereby promoting NF-κB activity [26]; phosphorylated ribosomal protein S6 kinase beta-1 (R70S6K (T389)), acting downstream of ERK and upstream of CREB1, transducing survival and proliferation signals and being an effector of TORC1 [27]; phosphorylated cyclic AMP-responsive element-binding protein 1 (CREB (S133)), a transcription factor known to co-regulate proliferation and that is activated by downstream kinases such as Akt and Rsk [28]; phosphorylated cellular tumor antigen p53 (P53 (S46)), where phosphorylation at S46 promotes the pro-apoptotic function of p53 [29]; and phosphorylated signal transducer and activator of transcription 3 (STAT3 (Y705)), a well characterized transcription factor acting downstream of MAPK and c-Src cascades, but best known for its involvement in the JAK-STAT signaling cascade [30].

**Fig. 3:**
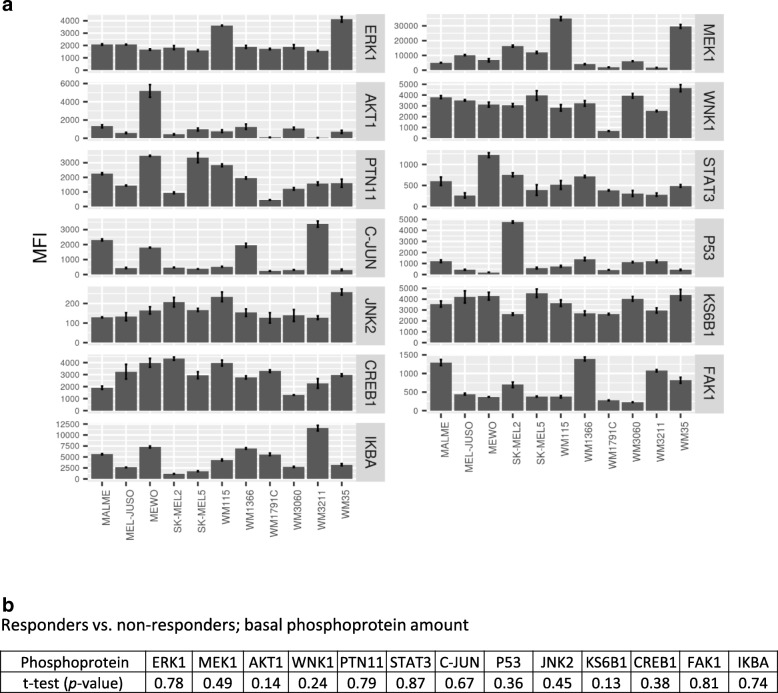
Basal phosphoprotein profiles of melanoma cell lines. **a** The phosphorylation status of 13 phospho-proteins was determined in 11 unstimulated melanoma cell lines using xMAP ELISA assays. Bar graphs represent median fluorescent intensity (MFI) analysed from *n* = 3 independently performed experiments (mean + SEM). **b** Statistical analysis comparing responders (SK-MEL-2, WM115 and WM3211) and non-responders was done using unpaired t-test

**Fig. 4:**
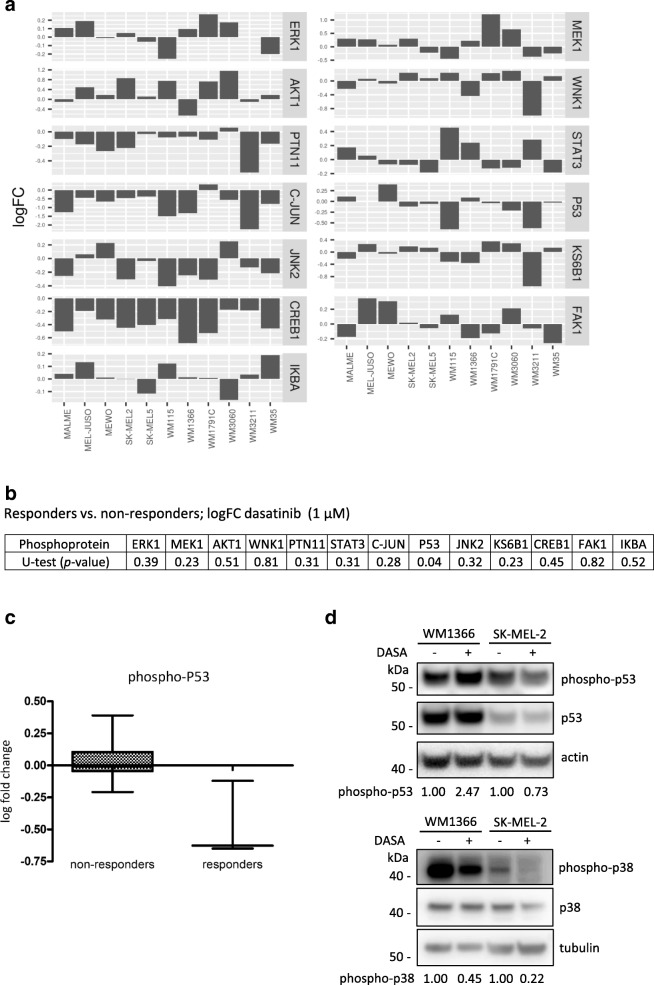
Treatment induced changes in phosphoprotein profiles in melanoma cell lines following dasatinib treatment. **a** The bar graphs represent the mean log fold changes of 13 phospho-proteins in 11 cell lines stimulated with 1 μM dasatinib, compared to unstimulated controls (DMSO), representative of *n* = 3 independent repeat experiments. **b** Statistical analysis comparing phosphoprotein changes between responders (SK-MEL-2, WM115 and WM3211) and non-responders using unpaired Mann Whitney U-test. **c** Post-treatment changes in phosphorylated p53 between responsive and non-responsive cell lines are shown as LogFC of p53(S46). **d** Western blots for total p53, phospho-p53 (S15), total p38 and phospho-p38 levels pre- and post-treatment with 1 μM dasatinib after 24 h. β-Actin and α-Tubulin served as loading controls. Quantifications of phospho-p53 and phospho-p38 are included below the respective blots

Basal phospho-protein expression across the melanoma cell line panel was highly heterogeneous (Fig. 3a). Comparing phospho-protein amounts between dasatinib responder cell lines (SK-MEL-2, WM115, WM3211) and resistant cell lines indicated that none of the tested phospho-proteins differed significantly between these two groups of cell lines (Fig. 3b). We therefore next studied the fold-changes of phospho-proteins in response to the addition of 1 μM dasatinib. As shown in Fig. 4a, the treatment induced changes were rather complex across the cell line panel, with both increases and decreases in various phosphoproteins observed as a consequence of dasatinib treatment. Comparing responder cell lines to dasatinib-resistant cell lines, we found that decreased amounts of phosphorylated p53 (S46) were associated with higher dasatinib responsiveness (Fig. 4b-c). The reduction of p53 phosphorylation was also detected when examining S15 phosphorylation in whole cell extracts of untreated and dasatinib-treated SK-MEL-2 responder cells but not, as expected, in WM1366 cells (Fig. 4d), indicating that this response might not be single-site specific. In both cell lines, dasatinib reduced the phosphorylation of p38, a kinase known to phosphorylate p53, with complete loss of p-p38 observed in SK-MEL-2 cells (Fig. 4d) Loss of p53 phosphorylation might therefore be an interesting candidate for a kinetic marker of dasatinib responsiveness in melanoma, pending more comprehensive validation in future studies.

## Discussion

Here, we studied the responsiveness of melanoma cells to dasatinib, alone or in combination with standard-of-care chemotherapeutic dacarbazine, by systematically analyzing growth inhibition and cell death at therapeutically meaningful concentrations ranges of both drugs. To our knowledge, this is the most comprehensive quantitative analysis of the effect of these therapeutics on melanoma cell proliferation and viability to date. Melanoma cells responded heterogeneously, with both dasatinib-responsive and highly dasatinib-resistant cell lines identified. Of thirteen phosphoproteins studied, all of which are directly or indirectly involved in regulating cell growth, proliferation, or cell cycle progression, none correlated with dasatinib responsiveness in their expression amounts. Interestingly though, the amounts of phosphorylated p53 (S46) dropped significantly in dasatinib-responsive cell lines following treatment, highlighting this response as a previously unknown kinetic marker candidate for susceptibility to dasatinib. Determining the specificity of such a marker more accurately, however, would require further studies, since p53 phosphorylation also dropped in one non-responsive cell line (WM3060).

Dasatinib has been studied comprehensively for its spectrum of kinase targets. Dasatinib inhibits diverse kinases besides the Src family at therapeutically meaningful concentration ranges. These targets include FAK, BTK, EPHs and p38 MAPK and others [3–7]. It therefore is understandable that dasatinib responsiveness can be difficult to predict from molecular signatures, since multiple targets and interacting pathways might play a context- and case-specific role. Correspondingly, studies conducted in association to dasatinib-based clinical trials in melanoma could not identify reliable predictors of dasatinib responsiveness. While the expression of Src family kinases appears to increase with disease progression in melanoma, high amounts of Src do not correlate with decreased survival in metastatic disease [17]. The expression of caveolin-1, a scaffolding protein that mediates the activation of Src family member FYN, was found to be increased in patients responding to dasatinib (*p* = 0.06), indicating that modulators of Src activity might provide scope for marker development [17]. c-KIT, which is rarely mutated in cutaneous melanoma but more frequently in acral and mucosal melanomas [31], can be inhibited by dasatinib in cultured cells with an IC50 of approximately 60–80 nM [3]. Within the cell line panel studied here, WM3211 cells harbor a c-KIT mutation, and we found this cell line to be dasatinib responsive. However, patients harboring c-KIT mutated melanoma did not uniformly respond to dasatinib in clinical trials [9, 10, 32]. Taken together, since a subgroup of melanoma patients appear to benefit from dasatinib-based treatments, the complexity of signaling processes affected by dasatinib might prevent reliable pre-treatment molecular markers from being developed.

## Conclusions

Instead of studying pre-treatment markers, an alternative approach lies within investigating if kinetic markers for dasatinib responsiveness can be identified. We found that the amounts of p53 (S46) drop upon dasatinib treatment in responsive melanoma cell lines. Among other kinases, the direct dasatinib target p38 MAPK can phosphorylate p53 at S46 [33]. p53 is a well known tumour suppressor that, upon activation, interferes with cell cycle progression and initiates apoptotic cell death. In contrast to many other cancers, p53 is rarely mutated in melanoma and instead is kept in check by other protein regulators that impair p53 activity or that limit p53 expression amounts [34, 35]. Therapeutically restoring p53 activity therefore is an innovative strategy in current translational melanoma research. The phosphorylation of p53 at position S46 is typically associated with the function of p53 as an apoptosis inducer [36, 37], so that the observed reduction in basal S46 phosphorylation in dasatinib responsive cell lines would likely blunt cell death-inducing responses that rely on p53-dependent gene transcription, in particular in combination treatment scenarios with genotoxic drugs such as dacarbazine. Indeed, preventing p53 S46 phosphorylation suppresses apoptosis responses [38]. We also found that de-phosphorylation might not be limited to the S46 site, since also S15 phosphorylation dropped following dasatinib treatment. This site is considered an initial nucleation site for subsequent additional p53 phosphorylation in response to DNA damage, but can also be found phosphorylated in untreated cells at steady state [39]. In our setting, this might contribute to explaining why impaired proliferation upon dasatinib treatment cannot effectively translate into cell death, and why dasatinib treatment did not synergize with dacarbazine. Our cell line-based study obviously has its limitations in that in vivo efficacy and consequences of dasatinib treatments cannot be captured. This becomes apparent when considering that tumours regressed, at least partially, in dasatinib-responsive patients [10], yet high amounts of cell death in response to dasatinib cannot be observed in vitro. How melanoma cells are eliminated in patients receiving dasatinib is presently unclear. Recent studies provide interesting data in relation to this question: Immune modulatory effects of dasatinib seems to contribute towards anti-tumour responses in vivo. For example, dasatinib decreased the amount of regulatory T cells and enhanced tumour infiltration by cytotoxic T cells in a mastocytoma tumour model [40]. In a colon cancer model, dasatinib inhibited tumour-associated myeloid cells and lead to apoptosis induction in Colo205 cells, whereas this cell line was largely resistant to dasatinib in vitro [41]. Nevertheless, the absence of synergies or potentiating effects between dasatinib and dacarbazine treatments should be taken into account when considering future clinical studies in melanoma patients in which dasatinib could be combined in a combination treatment settings with DNA alkylating drugs or other genotoxic therapeutics that induce p53 dependent apoptosis.

## Methods

### Cell culture, reagents and drug treatments

Cells were obtained as authenticated stocks from the ATCC (VA, USA) (WM115, SK-MEL-1, SK-MEL-5, Malme-3 M, MeWo, SK-MEL-2), the Wistar Institute (PA, USA) (WM35, WM3211, WM1366, WM1791C, WM3060) and the DSMZ (Deutsche Sammlung von Mikroorganismen und Zellkulturen GmbH, Germany) (Mel Juso). Five cell lines carried activating BRAF mutations (WM115, WM35, SK-MEL-5, SK-MEL-1, Malme-3 M), four cell lines NRAS mutations (Mel Juso, WM3060, SK-MEL-2, WM1366), one cell line a c-KIT mutation (WM3211), and two cell lines were BRAF/NRAS/c-KIT wildtype (WM1791C, MeWo). Cells were cultured in DMEM (Lonza, Slough, UK) supplemented with 4 mM L-glutamine, 4.5 g/l glucose, 10% (*w*/*v*) heat inactivated fetal bovine serum (Sigma-Aldrich), 100 U/ml penicillin and 100 mg/ml streptomycin (Sigma-Aldrich). Cells were grown at 5% CO_2_ and 37 °C. To study treatment responses, cells were seeded into flat bottom 96 well plates and treated with dasatinib (LC Laboratories, MA, USA), dacarbazine (Medac GmbH, Germany) or combinations of both drugs. Drugs were added simultaneously.

### High throughput cell proliferation and cell death measurements

For proliferation measurements, cell numbers were flow cytometrically determined by fixed volume event counts. For cell death measurements, cells were stained with propidium iodide after 72 h of treatment (final concentration = 1.3 μg/mL). Measurements were performed on a BD LSRII SORP HTS cytometer (BD Biosciences, NY, USA). Data files were exported in .fcs format and analyzed using Flowing Software (Turku Centre for Biotechnology, Finland). All experiments were independently repeated at least three times and all treatment conditions were measured in triplicates.

### Luminex xMAP technology measurements

Cells were seeded into 96 well plates and lysed after 24 h treatment with dasatinib. The protein concentrations in cell lysates were adjusted to 300 μg/ml, using the BCA assay kit for protein concentration determination (Pierce). xMAP bead-based ELISA type assays (Luminex xMAP assay, TX, USA) were performed on a Luminex FLEXMAP 3D® instrument using a custom developed phosphoprotein 13-plex panel (ProtATonce, Athens, Greece): RAC-alpha serine/threonine-protein kinase (AKT) - S473, Focal adhesion kinase 1 (FAK1) - Y397, Transcription factor AP-1 (c-JUN) - S63, dual specificity mitogen-activated protein kinase kinase-1 (MEK1/MP2K1) - S217/S221, Mitogen-activated protein kinase-3 (ERK1/MK03) - T202/Y204, Mitogen-activated protein kinase 9 (JNK2/MK09) - T202/Y204, Tyrosine-protein phosphatase non-receptor type 11 (PTN11/SHP2) - Y542, Cyclic AMP-responsive element-binding protein 1 (CREB) - S133, NF-κB inhibitor alpha (IκBa) - S32/S36, Ribosomal protein S6 kinase beta-1 (KS6B1/P70S6K) - T389, Cellular tumor antigen p53 (P53) - S46, Signal transducer and activator of transcription 3 (STAT3) - Y705, Serine/threonine-protein kinase WNK1 (WNK) - T60. For normalization, the levels of total GAPDH protein were analyzed.

### Western blot analysis

Melanoma cell lines were homogenized in lysis buffer (150 mM NaCl, 1 mM EDTA, 20 mM TRIS, 1% Triton X-100, pH = 7.6) with addition of PhosSTOP phosphatase inhibitor cocktail and cOmplete protease inhibitor cocktail (Roche, Switzerland). Total protein concentrations of samples were determined by Bradford protein assay (Roti-Quant, Carl Roth GmbH, Germany). Samples were loaded (30 μg) and separated on Bolt 4–12% Bis-Tris Plus Gels (Invitrogen, CA, USA) at 150 V, 400 mA for 40 min. Proteins were transferred to nitrocellulose membranes using the iBlot 2 gel transfer device (Thermo Fisher Scientific, MA, USA). The membranes were blocked in 5% BSA in TBST for 1 h at room temperature and incubated with primary antibodies overnight at 4 °C. The following primary antibodies were used: rabbit-anti p53 (1:1000, CST, MA, USA), anti-mouse beta-Actin (1:1000, CST, MA, USA) and rabbit-anti phospho-p53 (S15) (1:1000, R&D Systems, MN, USA), anti-rabbit p38 MAPK (1:1000, CST, MA, USA), anti-mouse phospho-p38 MAPK (T180 / Y182) (1:1000, CST, MA, USA), anti-mouse alpha-Tubulin (1:10000, CST, MA, USA),. The membranes were washed 3 × 10 min in TBST and incubated with secondary antibody HRP goat-anti rabbit (1,5000) for 1 h on a room temperature followed by 3 × 10 min wash in TBST. Luminata Forte Western HRP substrate (Merck Chemicals GmbH, Germany) was added and chemiluminescence signal was detected on Amersham Imager 600 system (GE Healthcare, IL, USA). Signal intensities above background were quantified using ImageJ software.

### Statistics

GraphPad Prism (GraphPad Software, CA, USA) was used for statistical analysis.

## Abbreviations

AKT1: RAC-alpha serine/threonine-protein kinase
BTK: Bruton’s tyrosine kinase
c-JUN: Transcription factor AP-1
CREB: Cyclic AMP-responsive element-binding protein 1
DTIC: Dacarbazine
EGFR: Epidermal growth factor receptor
EPHs: Various ephrin receptors
ERK1: Mitogen-activated protein kinase-3
FAK1: Focal adhesion kinase 1
IκBα: NF-κB inhibitor alpha
JNK2: Mitogen-activated protein kinase 9
MEK1: Mitogen-activated protein kinase kinase-1
ORR: Objective response rate
p38 MAPK: p38 mitogen-activated protein kinase
P53: Cellular tumor antigen p53
PFS: Progression-free survival
PTN11: Tyrosine-protein phosphatase non-receptor type 11
R70S6K: Ribosomal protein S6 kinase beta-1
STAT3: Signal transducer and activator of transcription 3
WNK: Serine/threonine-protein kinase WNK1
